# Large bladder diverticulum causing direct extrinsic compression of the left ureter

**DOI:** 10.1016/j.eucr.2024.102779

**Published:** 2024-06-22

**Authors:** Shravankrishna Ananthapadmanabhan, Zoe Williams, Henry Wang, Nicola Jeffery, Nicholas Mehan

**Affiliations:** a Nepean Urology Research Group, Nepean Hospital, Derby St, Kingswood, New South Wales, 2747, Australia; b University of Sydney, Sydney Medical School, Camperdown, New South Wales, 2050, Australia

**Keywords:** Bladder diverticulum, Bladder neck obstruction, Bladder neck incision, Hydroureter

## Abstract

Bladder diverticula are herniations of the bladder mucosa through the muscular layer and can be congenital or acquired. Acquired bladder diverticula are almost always associated with bladder outlet obstruction. Bladder diverticula are uncommon and often asymptomatic, however, can present with non-specific lower urinary tract symptoms, haematuria, or urinary tract infection. We report a rare case of a large bladder diverticulum causing extrinsic left ureteric compression in a 37-year-old male with a high bladder neck presenting as left flank pain and hydronephrosis. A bladder neck incision successfully resolved voiding symptoms and decompressed the diverticulum leading to resolution of ureteric obstruction.

## Introduction

Bladder diverticula represent herniation of the bladder mucosa through the muscular layer.^1^ They may be congenital, occurring in children with congenital deficiency in the muscularis propria layer, or acquired in association with bladder outlet obstruction. It is estimated that up to 15 % of adult patients with lower urinary tract obstruction have bladder diverticula, though they may be asymptomatic and are often diagnosed incidentally.^2^

Congenital bladder diverticula are associated with genetic syndromes affecting the connective tissues such as Ehlers-Danlos syndrome and Williams' syndrome. They are hypothesised to arise due to embryological failure of detrusor muscle formation or detrusor hypoplasia. Most congenital diverticula are located in close proximity to the ureter where the bladder wall is weakest. This location predisposes to vesicoureteral reflux and hydronephrosis.^3^ In contrast, acquired bladder diverticula are secondary to bladder outlet obstruction causing raised intra-vesical pressures which herniates the bladder mucosa through detrusor muscle bundles.^4^ Adult bladder diverticula typically occur posterolaterally.

Deficiency of muscle fibres in the diverticulum wall results in urinary stasis and subsequently urinary tract infections and bladder stone formation.^1^^,^^5^ Chronic inflammatory change within the diverticulum caused by stagnant urine also represents a risk factor for malignancy.^6^ In rare cases, large bladder diverticula can also exert mass effect on abdominal organs, with reports describing symptoms including epigastric discomfort, dyspepsia, as well as diagnoses of mechanical bowel obstruction.^7^^,^^8^

We present the case of a 37-year-old male who was investigated for left flank pain, on a background of long-standing lower urinary tract symptoms (LUTS). CT imaging demonstrated a large left-sided bladder diverticulum which projected posteriorly onto the left ureter. Severe hydroureteronephrosis (HUN) was evident proximal to this point of contact. Video urodynamic studies confirmed high pressure voiding, however, with absence of left vesico-ureteric reflux. To our knowledge, this is the second documented case of a large bladder diverticulum causing direct ureteric compression and obstruction.

## Case description

A 37-year-old male was referred to a urologist with a 2-week history of left sided flank pain. This was on a background of longstanding voiding symptoms including slow stream and incomplete emptying, although bother related to these lower urinary tract symptoms (LUTS) was minimal. He had been diagnosed with one episode of urinary tract infection the year prior. He had no other significant medical or surgical history.

A renal tract ultrasound and subsequent CT Urogram demonstrated a trabeculated bladder, and a 10cm diverticulum arising from the left posterior wall. The diverticulum extended superiorly and posteriorly with mass effect onto the left ureter. Distal to the point of contact, the ureter calibre was normal, however proximally, moderate-severe hydroureteronephrosis (HUN) was present and suggestive of extrinsic compression of the left ureter (Fig. A, Fig. B A and B).

**Fig. A:**
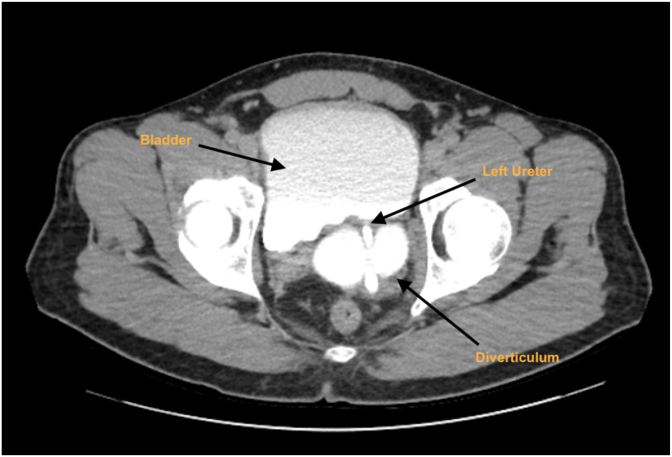
Computer tomography intravenous pyelogram (CT IVP) axial slice demonstrating direct compression of the left ureter by large bladder diverticulum.

**Fig. B:**
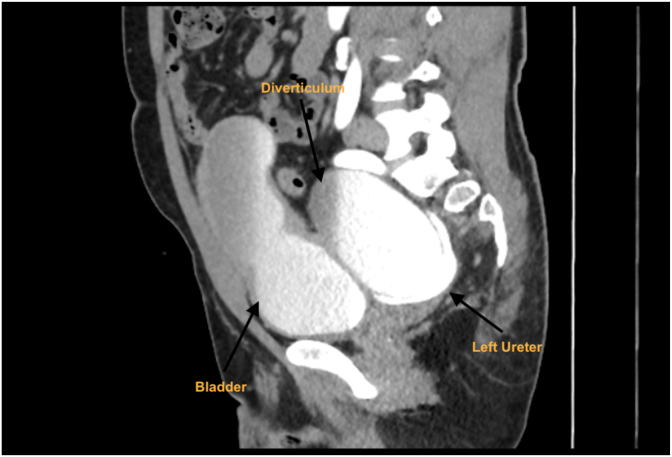
Computer tomography intravenous pyelogram (CT IVP) sagittal slice demonstrating direct compression of the left ureter by large bladder diverticulum.

To assess voiding function and possible vesico-ureteric reflux, a video urodynamic study was performed (UDS, Fig. C). The study revealed a maximum flow rate (Qmax) of 8 mL/second and a detrusor pressure at maximum flow (pDetQmax) of 80, with a calculated bladder outlet obstruction index (BOOI) of 64. Post-void residual volumes were notably elevated at 550mL. Compliance was normal to 450mL but was mildly decreased when approaching maximal bladder capacity of 800mL (14mL/cm H2O). **The diverticulum did not empty on voiding and no vesico-ureteric reflux was observed throughout the UDS**.

**Fig. C:**
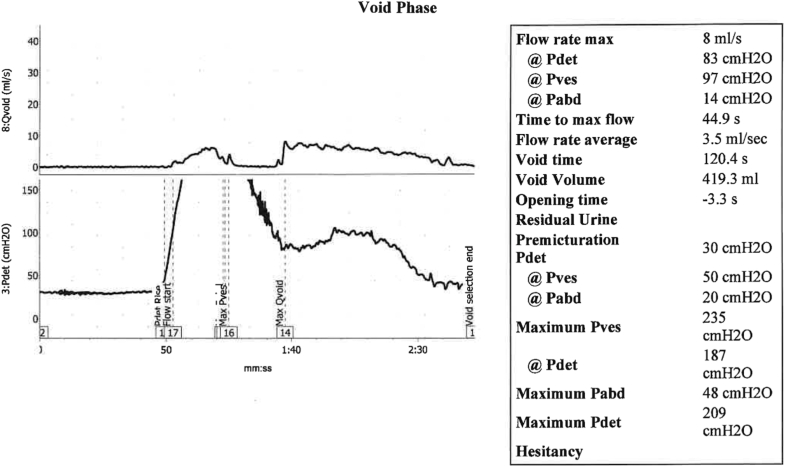
Trace of voiding phase of urodynamics (UDS) study.

A cystoscopy was performed, demonstrating a small, non-occlusive prostate, with a high bladder neck. The bladder was trabeculated, and the large bladder diverticulum was seen to arise along the left posterolateral wall, posterior to the left ureteric orifice. The left ureter was morphologically normal. A left retrograde pyelogram performed showed severe HUN proximal to the distal ureter, with suboptimal drainage under fluoroscopic and direct visualisation.

Treatment options were discussed including the risk of retrograde ejaculation associated with bladder neck incision. Having completed his family and considering all options, the patent proceeded with a bladder neck incision. **Using a monopolar Collin’s knife, deep incisions were made down to circular muscle fibres at the 5 and 7 o’-clock positions**. Following this the patient experienced significant improvement in LUTS and resolution of left flank pain. Repeat flow studies demonstrated an improvement of Qmax to 33mL/second. Post-void residuals had also significantly decreased, down to 51mL. A Diethylenetriamine Pentaacetate (DTPA) nuclear medicine study was performed to assess for obstruction, with a borderline but reassuring T1/2 of 11 minutes (Fig. D). No urinary incontinence or retrograde ejaculation was reported at follow-up. Although a diverticulectomy was offered, given the significant improvement in voiding function and resolution of symptoms, the patient opted to proceed with expectant management.

**Fig. D:**
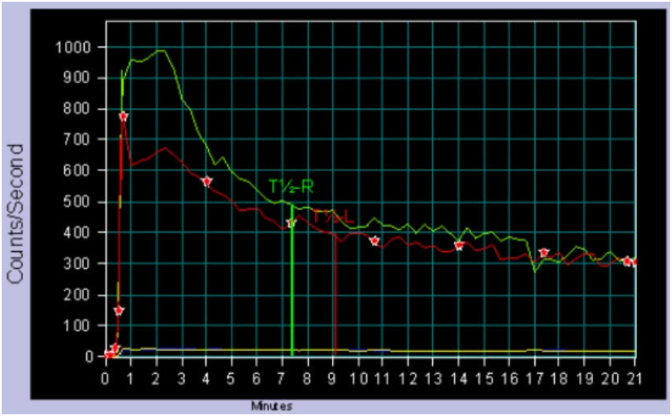
Post bladder neck incision (BNI) diethylenetriamine Pentaacetate (DTPA) curve.

## Discussion

Bladder diverticula represent herniation of the bladder mucosa through detrusor muscle fibres. It is an uncommon pathology and can be categorised as congenital or acquired.^9^ An estimated 15 % of adults with obstructive lower urinary tract conditions have bladder diverticular disease, with a predilection for males aged over 60 years.^2^ Management considerations discussed in this case includes bladder outlet obstruction in the setting of a large bladder diverticulum, and the resulting ureteric obstruction.

Bladder diverticula management is dependent upon patient symptom burden, and the presence of complications. Most diverticula are asymptomatic, discovered incidentally, and can be managed conservatively. When symptomatic, patients often experience LUTS, haematuria and urinary tract infections. Additionally, urinary stasis within diverticula can predispose patients to stone formation, and chronic inflammation can lead to bladder cancers. When intervention is deemed necessary, it is important to treat the cause of the underlying BOO first, prior to reassessing symptomatology.^10^ Options for treatment of diverticula include endoscopic procedures such as fulguration of the diverticulum mucosa, and surgical resection with a diverticulectomy.^2^^,^^10^^,^^11^

High bladder neck or bladder neck elevation (BNE) can be obstructive in nature. BNE can be quantified by measuring the prostatic urethral angulation (PUA). Increasing PUA is believed to result in increased energy loss when voiding, resulting in decreased urine velocity in an inverse relationship.^12^ Hou et al. demonstrated that greater PUA was negatively correlated with International Prostate Symptom Score (IPSS) and Qmax.^13^ Additionally, Ku et al. also found higher PUA to be associated with higher pDetQMax and BOOI. Standard of care for surgical intervention of BNE involves transurethral resection, or bladder neck incision (BNI). A significant concern relating to these procedures in young males is the potential for side effects such as retrograde ejaculation and urinary incontinence. Reassuringly however, in a retrospective series of superficial BNI performed in 37 male pediatric and adolescent patients, all cases reported antegrade ejaculation, and only 2 (5.4 %) experienced moderate incontinence. Excellent functional outcomes have also been demonstrated in our patient, with no incontinence, preservation of antegrade ejaculation, resolution of clinical symptoms, and significant improvements in functional voiding parameters on follow-up.

Management of the ureteric obstruction in this case aligns with standard care for extrinsic compression. The differential of vesico-urethral reflux (VUR) as the cause of the HUN was investigated with the use of a video UDS, which ruled out VUR. Although considered for a ureteric stent at time of initial cystoscopy, a retrograde pyelogram performed visualised efflux from the left ureteric orifice, and contrast clearance was seen to clear on fluoroscopy. Following BNI, we surmise that the significant reductions in PVR observed, correlate with decreased diverticulum size, resulting in resolution of ureteric obstruction.

Bladder diverticula resulting in ureteric obstruction has been rarely reported in the literature,.^14^^,^^15^ This case highlights a number of related pathologies stemming from BOO. From our literature review, this is the second documented case of ureteric obstruction related to extrinsic compression from a bladder diverticulum. We demonstrate the utilisation of BNI to treat BNE, which successfully resolved both BOO and left ureteric obstruction.

## Conclusion

Ureteric obstruction secondary to extrinsic compression from a bladder diverticulum is an exceedingly rare pathology within the literature. In line with bladder diverticulum management principles, the underlying bladder outlet obstruction was treated with the use of a bladder neck incision. Following treatment, significant improvements in voiding function was observed, in conjunction with resolution of clinical symptoms. Decreased diverticulum size relating to the improved voiding function also led to the resolution of left ureteric obstruction.

## Statement of ethics

Informed patient consent has been obtained for publication of this case report and clinical images.

## CRediT authorship contribution statement

**Shravankrishna Ananthapadmanabhan:** Writing – original draft. **Zoe Williams:** Writing – review & editing. **Henry Wang:** Writing – review & editing, Conceptualization. **Nicola Jeffery:** Conceptualization. **Nicholas Mehan:** Supervision, Conceptualization.

## Declaration of competing interest

The authors have no conflicts of interest to declare.

## References

[1] Burns E. (1944). Diverticula of the urinary bladder. Ann Surg.

[2] Geavlete P.A., Georgescu D., Drăguţescu M., Geavlete B. (2016). Endoscopic approach to bladder diverticula. Endosc Diagn Treatm Urin Blad Pathol.

[3] Psutka S.P., Cendron M. (2012 Apr). Bladder diverticula in children. J Pediatr Urol.

[4] Miller A. (1958 Mar). The aetiology and treatment of diverticulum of the bladder. Br J Urol.

[5] Cox L., Rovner E.S., Partin A.W., Peters C., Kavoussi L.R. (2020). Campbell-walsh Urology 12th Edition Review.

[6] Whitefield B. (2010). Urinary bladder diverticulum and its association with malignancy: an anatomical study on cadavers. Rom J Morphol Embryol.

[7] Kumar S., Jayant K., Barapatra Y., Rani J., Agrawal S. (2014). Giant urinary bladder diverticula presenting as epigastric mass and dyspepsia. Nephro-Urol Mon.

[8] Akbulut S., Cakabay B., Sezgin A., Isen K., Senol A. (2009). Giant vesical diverticulum: a rare cause of defecation disturbance. World J Gastroenterol.

[9] Sengupta S., Basu S., Gupta S. (2020 Nov 23). An insight into the management of urinary bladder diverticulum: a retrospective observational study. Int J Adv Med.

[10] Ho M.C., Hashim H. (2022). Surveillance and management of bladder diverticulum in the setting of bladder outlet obstruction. Curr Blad Dysfunc Rep.

[11] Pham K.N., Jeldress C., Hefty T., Corman J.M. (2016). Endoscopic management of bladder diverticula. Rev Urol.

[12] Cho K.S., Kim J., Choi Y.D., Kim J.H., Hong S.J. (2008). The overlooked cause of benign prostatic hyperplasia: prostatic urethral angulation. Med Hypotheses.

[13] Hou C.-P., Lin Y.-H., Chen C.-L., Tsai Y.-L., Chang P.-L., Tsui K.-H. (2016). Impact of the static prostatic urethral angle on men with lower urinary tract symptoms. Urol Sci.

[14] de Dieu Tumusifu Manegabe J., Nteranya D.S., Balemba G.M. (2022). Multiple bladder diverticula presenting in an 82-year-old Congolese male. Case Rep Surg.

[15] Kim S., Park S.H., Kim D.Y., Yun S.J., Lee O.J., Han H.S. (2018). Bilateral obstructive uropathy caused by congenital bladder diverticulum presenting as hypertensive retinopathy. J Korean Med Sci.

